# Correlation between perioperative surgical factors and implant-related complications in the basicervical fractures of femoral neck treated with proximal femoral nail antirotation: a retrospective review study in 149 elderly patients

**DOI:** 10.1007/s00590-026-04812-z

**Published:** 2026-07-13

**Authors:** Paphon Sa-ngasoongsong, Nattawat Angkavanich, Konlawat Sabsuantang, Norachart Sirisreetreerux, Nachapan Pengrung, Noratep Kulachote

**Affiliations:** 1 Department of Orthopaedics, Faculty of Medicine Ramathibodi Hospital, Mahidol University, Bangkok, Thailand; 2 Phrapokklao Hospital, Chanthaburi, Thailand

**Keywords:** Basicervical femoral neck fracture, Mechanical complication, Fixation failure, PFNA, Risk factors, Cephalomedullary nail

## Abstract

**Purpose:**

Basicervical femoral neck fractures (BFNFs) are uncommon in the elderly but associated with higher postoperative complications compared to intertrochanteric fractures. Proximal femoral nail antirotation (PFNA) is one of the preferred surgical options for intertrochanteric fracture due to its minimal invasiveness, fewer complications, and better in postoperative functional outcomes. However, limited research addressed the outcomes and the risk factors for implant-related complications after using PFNA.

**Methods:**

This retrospective study included 149 patients treated with PFNA fixation in a University hospital. Inclusion criteria were age over 65 years, BFNFs from low-energy trauma, prefracture ambulatory level as independent ambulation with or without gait aids, with available data and follow-up period of at least 1 year. Exclusion criteria included open fracture, pathological fracture, multiple or high-energy traumas, and open fractures. Implant-related complications was defined as nail breakage, peri-implant fracture, varus collapse, blade cut-out or cut-through, and excessive sliding (> 10 mm). Demographic, clinical, and radiographic data were collected and analyzed. Multivariate logistic regression analysis was used to identify the risk factors.

**Results:**

Of the 149 patients (26 males, 123 females) with an average age of 81 ± 9 years. Seventeen patients (11.4%) had mechanical complication and 11 patietns (7.4%) had reoperation. Univariate analysis showed that BMI, ASA grade 4, diabetes, tip-apex distance (TAD), calcar-referenced TAD, neck shaft angle, lateral angulation, anteromedial cortical support (AMCS) reduction, and Chang reduction quality criteria were potential predictors. Following multivariate analysis, only BMI, ASA grade 4, lateral angulation and AMCS were significant risk factors for implant-related complication (*p* < 0.05 all).

**Conclusion:**

The elderly patients with BFNFs and treated with PFNA are generally safe with acceptable reoperation rate. However, the proper reduction technique using AMCS and lateral angulation are essential for successful outcomes. Moreover, the patients with high BMI and ASA grade 4 are significantly associated with implant failure and required special attention.

## Background

Basicervical femoral neck fractures (BFNFs) are a type of femoral neck fractures that involves the junction of the femoral neck and intertrochanteric region. These fractures are uncommon as accounting for 1.8% of proxiaml femoral fractures [1, 2] and often classified as extracapsular fracture. However, due to the anatomical location of these fractures and lack of cancellous interdigition compared to intertrochanteric area [3], previous studies found that the postoperative complications such as nonunion, blade cut-out, cut-through, excessive blade sliding, avascular necrosis of the femoral head, and secondary osteoarthritis are upto 16% [4] and more common compared to intertrochanteric fractures [2, 5, 6].

Generally, the surgical treatment of BFNFs is similar to those in the intertrochanteric fractures with many types of fixation devices, such as dynamic hip screw or cephalomedullary nail. Among these surgical options, proxiaml femoral nail antirotaton (PFNA) is one of the most preferred cephalomedullary nail due to its minimally invasive surgical techqniue with shorter operative time, reduced blood loss, shorter hospital stays, and the benefits of earlier postoperative weight-bearing and mobilization [5, 7, 8]. However, the data on using PFNA in BFNFs related to the success rate and postoperative complications are still lacking. Moreover, to our knowledge, the correlation between the PFNA-related complication and BFNFs has not been established.

Therefore, this study aims to evaluate postoperative surgical outcomes in the elderly patients sustained BFNFs and treated with PFNA, and to identify the risk factors for implant-relaed complication in these patients.

## Materials and methods

### Study design, participants, and inclusion and exclusion criteria

The present study was designed as a single-center retrospective review study in the elderly patients who sustained BFNFs and treated with PFNA fixation in Faculty of Medicine Ramathibodi Hospital, Mahidol University between January 2005 and August 2023. Prior approval was obtained from the institutional review board (COA. No. MURA2023/861). The inclusion criteria were the paitents who 1) aged over 65 years, 2) sustained BFNFs not older than 3 weeks from low-energy trauma defined by the definition from Watson et al. [9], 3) had pre-fracture ambulatory level classified as independent ambulation with or without gait aids [10], 4) had been treated with PFNA, and 5) had available clinical notes and adequate preoperative and postoperative radiographs with at least 1 year of follow-up data. The exclusion criteria were open fracture, pathologic fracture other than osteoporotic fracture, history of prior fracture or surgery, and multiple fractures from high-energy trauma.

### Surgical procedure and postoperative protocol

Following optimization of the patient’s medical condition and preoperative planning, surgery was generally performed within 48 h of admission. All operations were performed by experienced orthopaedic trauma surgeon who had performed hip fracture surgery more than 5 years. The fractures were initially reduced on a traction table with closed reduction methods. If the fracture reduction was unacceptable, mini-open or open reduciton techniques would be used until the fracture alignment was accepted. After successful reduction, a PFNA implant (Depuy Synthes, Oberdorf, Switzerland), in size 9 to 11, was chosen based on the intraoperative measurement and then inserted with a minimally invasive technique with one static distal locking screw, as previously reported [11, 12]. Cement augmentation with a 3 to 4 mL polymethyl methacrylate (PMMA)-based cement (Traumacem V + , Depuy Synthes, Zuchwil, Switzerland) was indicated and performed under image intensifier using the standard technique [13], in the patients with unstable fractures and having severe osteoporosis [14].

Postoperatively, All patients were treated with the same postoperative rehabilitation protocol. An intermittent pneumatic compression device was used for deep vein thrombosis prophylaxis, and pharmacological anticoagulants were administered in selected high-risk cases. All patients were mobilized within 24 h after surgery and allowed to perform to weight bering as tolerated exercise on the injured hip with a walker. Radiographic and clinical follow-ups were scheduled at 2 weeks, 6 weeks, 3 months, 6 months, and 1 year postoperatively.

### Data collection and outcome measurement

**Fig. 1:**
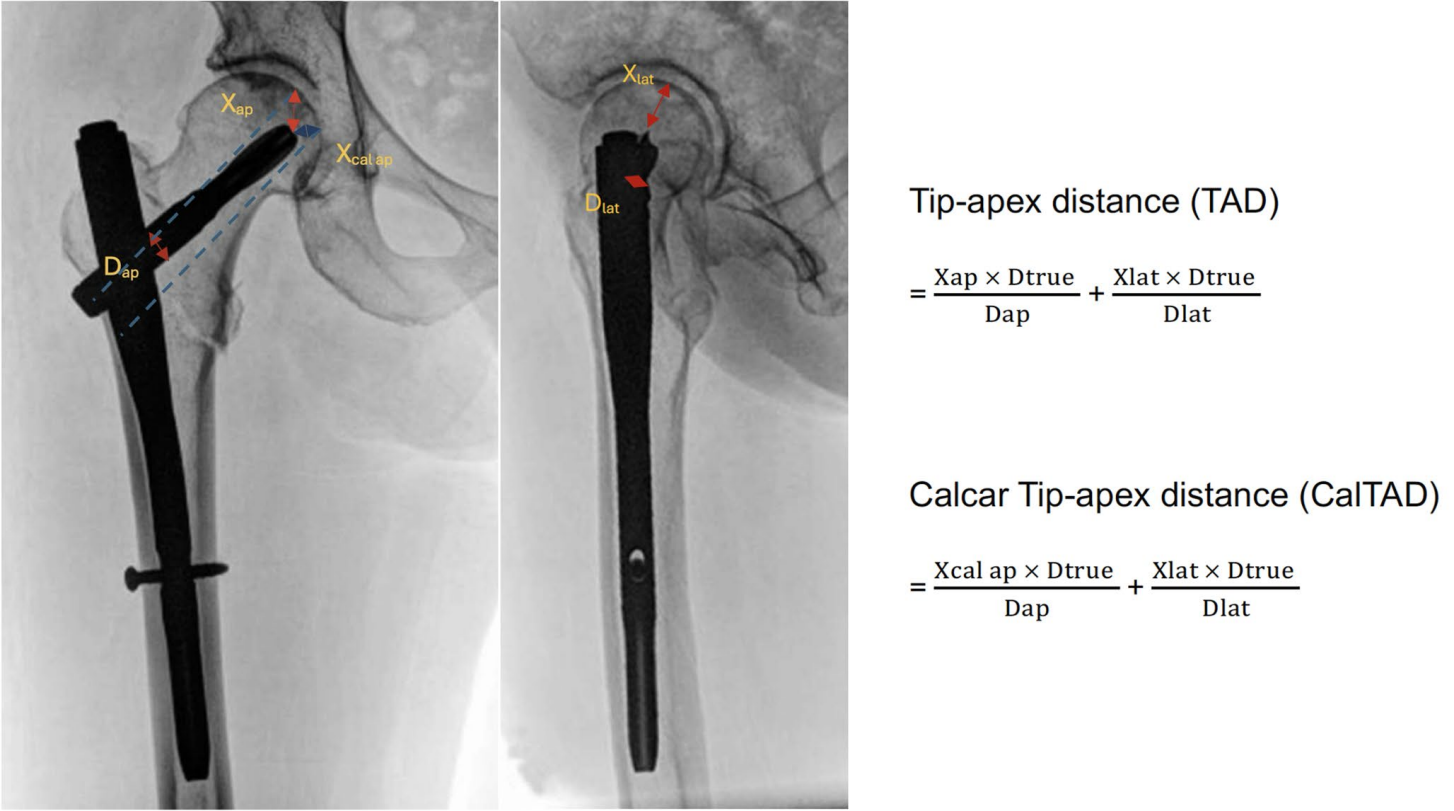
Tip-apex distance (1) and calcar tip-apex distance (CalTAD) measurement on anteroposterior and lateral radiographs. Both are the sums of distance from helical blade tip to the apex of femoral head using femoral neck axis (Xap) or to the line parallel and adjacent to medical cortex (Xcal ap) in anteroposterior view and lateral view (Xlat), and having correction for magnification by the ratio of true nail diameter (Dtrue) and measured nail diameter (Dap or Dlat)

**Fig. 2:**
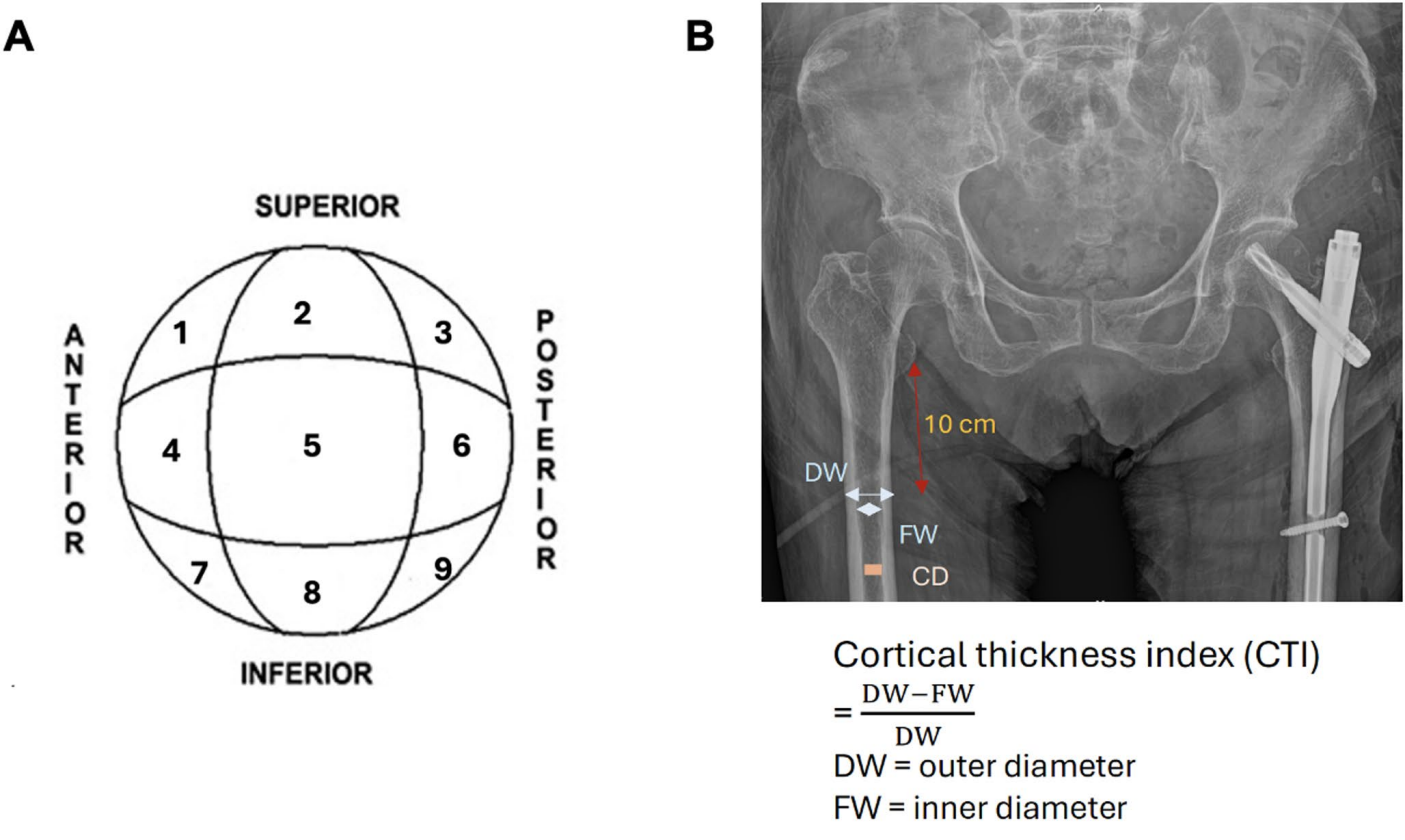
**A** The Cleveland 9 zones (**A**) for determining blade postion by dividing the femoral head into thirds on both anteroposterior and lateral view resultig in 9 distinct quadrants. **B** Cortical thickness index (CTI), measuring at 10 cm level below the lesser trochanter and calculated by measuring the ratio of the cortex thickness (DW-FW) to the total diameter of the femoral shaft (DW)

**Fig. 3:**
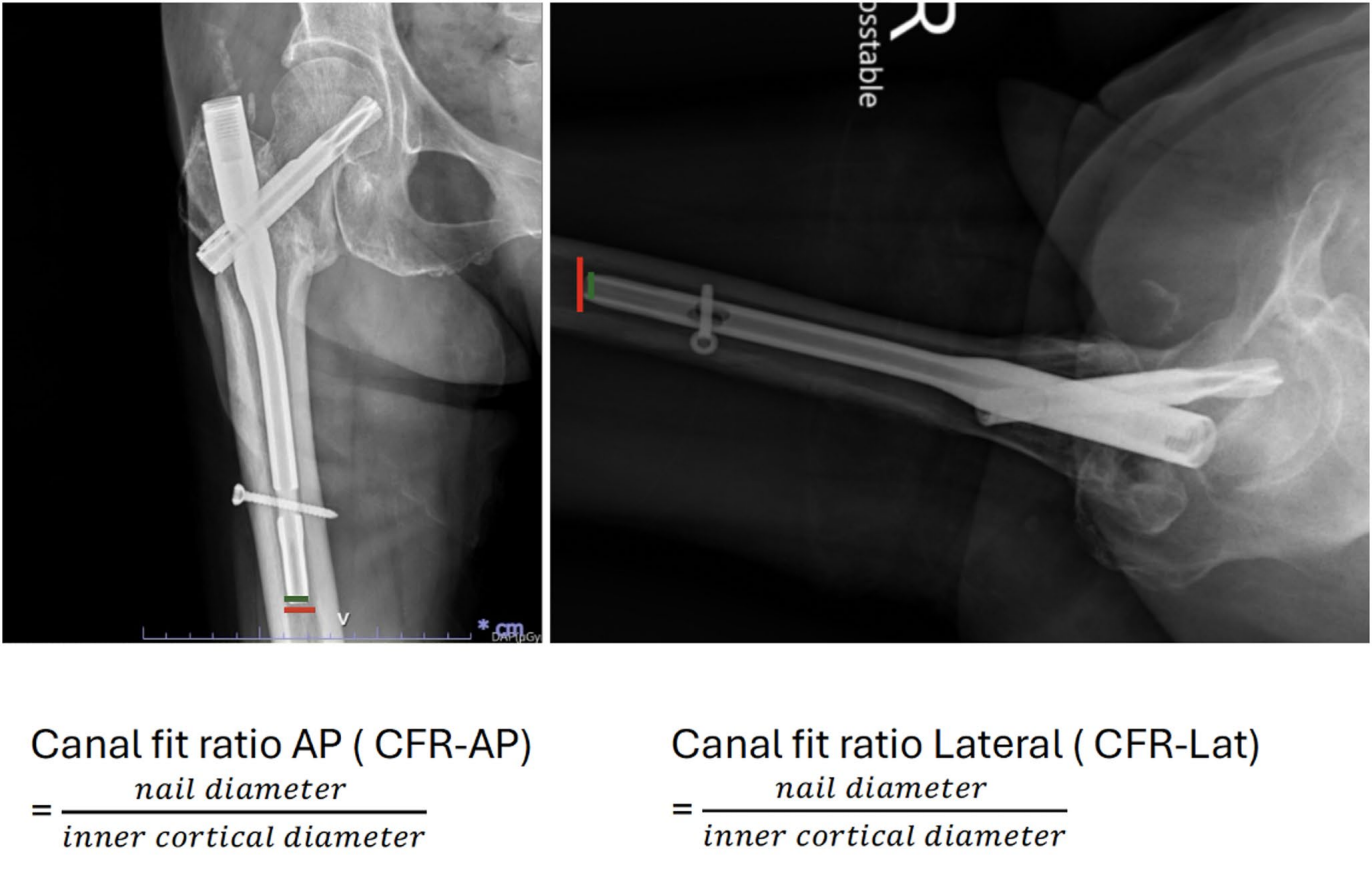
Canal fit ratio measurement as using the nail diameter compared to the narrowest part of medullary canal of femoral shaft in both anteroposterior (CFR-AP) and in lateral (CFR-Lat) views

The demographic data, including age, gender, body mass index (BMI), fracture side, American Society of Anesthesiologists (ASA) physical status, and comorbidities, were collected. Preoperative and postoperative radiogaphs were independently reviewed by two authors (P.S. and N.A.) with assessments repeated after one-month interval to determine intraobserver and interobserver reliability. The following radiographic parameters were recorded: tip apex distance [15, 16], calcar-referenced TAD (Cal-TAD) [17] (Fig. 1), blade position according to the Cleveland 9 zones [18], cortical thickness index (CTI) [19, 20] (Fig. 2), canal fit ratio in anteroposterior (CFR-AP) and lateral radiographs (CFR-lat) [21] (Fig. 3), neck shaft angle (NSA) [22], lateral angulation [23], positive anteromedial cortical support (AMCS) [24], Chang reduction quality criteria (CRQC) [25], and blade sliding distance [26]. Data related to postoperative implant-related complications, as defined by implant failure or breakage, peri-implant fracture, varus collapse, blade cut-out, blade cut-through with or without head perforation, and excessive sliding more than 10 mm [27], were collected. Reoperation and type of reoperation were also collected.

### Statistical analysis

Continuous data with normal distribution were summarized as means and standard deviations and compared using t-tests. Categorical data were presented as proportions and compared using Fisher’s exact test. Intraobserver and interobserver reliability were assessed by intraclass correlation (ICC) and Kappa statistic, for continuous and categorical variables, respectively. Disagreement in reliability between observers was managed by averaging the first measurements (continuous data) or consensus discussion (categorical data).

To assess risk factors for implant-related complications, comparisons were made between “No implant failure” and “Mechanical complication” groups. Both univariate and multivariable logistic regression analyses were performed to determine associations between risk factors and outcomes. A *p*-value < 0.15 was used to identify potential predictive factors, with statistical significance defined as *p* < 0.05. Data analysis was carried out using MedCalc software.

## Results

### Demographic data

During the study period, 642 elderly patients suspected BFNFs or intertrochanteric fractures and had been treated with PFNA were recruited. From these, 493 cases were excluded due to intertrochanteric fracture (*n* = 485), delayed presentation more than 3 weeks (*n* = 1), did not have 1-year available follow up data (*n* = 5), and multiple fractures from high-energy trauma (n-2). Therefore, a total of 149 patients was eligible for the analysis. The average age was 80.9 ± 9.1 years and 123 of them (82.6%) were female. The average BMI was 22.0 ± 4.5 kg/m^2^. Twenty-three patients (18.8%) were classified as ASA physical status grade 4. Following PFNA fixation, 17 patients (11.4%) had implant-related complications and were allocated into “Mechanical complication” (14 excessive sliding, 1 cut through, 1 varus collapse with nonunion, 1 varus collapse), while 132 patients (88.6%) were allocated into “No implant failure” group (Table 1). Regarding to the reoperation, a total of 11 patients (7.4%) required reoperation due to postoperative complications (8 in Mechanical complication group and 3 in No implant failure group). In Mechanical complciaiton group, the reoperations were performed as salvage bipolar hip replacement (*n* = 5) or revision surgery (*n* = 3). For No implant failure group, the reoperations were performed as total hip replacment (*n* = 2, due to 1 avascular necrosis of femoral head and 1 hip osteoarthritis) or resection arthroplasty (*n* = 1, due to infection). Table 1 showed comparison of demographic data between Mechanical complication (*n* = 17) and No implant failure (*n* = 132) groups. Mechanical complication group demonstraed a significant higher in median BMI (24.6 vs. 21.4 kg/m^2^) and proportion of ASA grade 4 (47.1 vs. 15.2%) than No implant failure group (*p* < 0.05 both). No significant difference was found in age, gender, comorbidities between groups (*p* > 0.05 all).

**Table 1:** Demogrphic data comparison between "No implant failure" and “Mechanical complcation” group

	No implant failure group (*n* = 132)	Mechanical complication group (*n* = 17)	*p*-value
Age, year^a^	79.3 ± 5.8	81.1 ± 9.4	0.43
Male:female^b^	22:110	4:13	0.50
BMI, kg/m^2c^	21.4 (18.6 to 24.1)	24.6 (19.9 to 28.0)	0.03*
Right:Left^b^	8:9	52:80	0.60
ASA class 4 (%)^d^	20 (15.2%)	8 (47.1%)	0.004*
*Comorbidity (%)*^d^			
Diabetes	47 (35.6%)	10 (58.8%)	0.11
Hypertension	102 (77.3%)	14 (82.3%)	0.76
Stroke	22 (16.7%)	1 (5.9%)	0.47
Cardiac disease	33 (25%)	4 (24%)	1.00
Renail failure	18 (13.6%)	4 (23.5%)	0.28

^a^Value presented as mean ± standard deviation

^b^Value presented as ratio

^c^Value presented as median (interquartile range)

^d^Value presented as number of case (percentage)

*Significant difference between groups with *p* < 0.05

### Postoperative radiographic parameters

Table 2 revealed the radiographic parameters in the initial postoperative film related to the PFNA fixation. The reliability of radiographic measurement demonstrated high intraobserver and interobserver reliabilty with ICC and Kappa statistic close to 0.9. No significant diffrence was found in CTI, canal dimater, CFR-AP, CFR-lat, and cement augementation use between both groups (*p* > 0.05 all). Mechanical complication group had significantly lower in mean NSA (133.5 vs. 137.0 degree) and proportion of positive AMCS (53 vs. 79%), compared to No implant failure group (*p* < 0.05 both). Mechanical complication group also showed non-statistically higher values in TAD, Cal-TAD, lateral angulation, and proportion of accepatble CRQC, compared to No implant failure group (*p* = 0.07, 0.06, 0.09, and 0.11).

**Table 2:** Radiographic parameters comparison between “No implant failure” and “Mechanical complcation” group

Radiographic parameters	No implant failure group (*n* = 132)	Mechanical complication group (*n* = 17)	*p*-value
TAD, mm^a^	23.9 ± 6.7	27.0 ± 8.1	0.07
Cal-TAD, mm^a^	24.0 ± 6.6	27.4 ± 8.2	0.06
Blade position^b^ IC or CC:other zones	107:25	12:5	0.31
CTI^a^	0.49 ± 0.10	0.47 ± 0.11	0.44
Canal diameter, mm^a^	15.1 ± 3.0	15.8 ± 3.5	0.40
CFR-AP^a^	0.76 ± 0.11	0.74 ± 0.11	0.43
CFR-lat^a^	0.57 ± 0.09	0.54 ± 0.11	0.29
NSA, degree^a^	137.0 ± 6.3	133.6 ± 9.0	0.05*
Lateral angulation, degree^a^	27.9 ± 5.9	30.5 ± 6.3	0.09
Positive AMCS^c^	104 (79%)	9 (53%)	0.03*
Initial postoperative CRQC acceptable:excellent^b^	47:85	10:7	0.11
Cement augmentation^c^	39 (30%)	5 (29%)	1.00

TAD, tip apex distance; Cal-TAD, calcar referenced tip apex distance; IC, inferior-center; CC, center-center; CTI, cortical thickness index; CFR-AP, canal fit ration in AP view; CFR-lat, canal fit ratio in lateral view; NSA, neck shaft angle; AMCS, anteromedial cortical support; CRQC, Chang reduction quality criteria

^a^Value presented as mean ± standard deviation,

^b^Value presented as ratio of cases with those conditions

^c^Value presented as number of cases (percentage)

*Significant diffence with *p* < 0.05

### Univariate and multivariate logistic regression analyses

Table 3 demonstrated univariate analysis (UVA) and multivariable analysis (MVA) for implant-related complication. After UVA, there were 8 potential predictors—BMI, ASA grade 4, TAD, Cal-TAD, NSA, lateral angulation, positive AMCS, and excellent grading in CRQC—that were included into MVA. However, MVA showed that only 4 predictors were revealed as the independent risk factors for implant-related complication as BMI (odds ration [OR] 1.18, 95% confidence interval [CI] 1.05 to 1.31, *p* = 0.004), ASA grade 4 (OR 4.98, 95% CI 1.72 to 14.43, *p* = 0.003), lateral angulation (OR 1.13, 95% CI 1.02 to 1.25, *p* = 0.02), and positive AMCS (OR 0.27, 95%CI 0.08 to 0.88, *p* = 0.03).

**Table 3:** Univariate and Multivariable analysis for fixation failure in basicivical femoral fractre threated with PFNA

	Univariate analysis		Multivariate analysis	
	OR (95% CI)	*p*-value	OR (95% CI)	*p*-value
Age	0.98 (0.93 to 1.03)	0.43		
Male	1.54 (0.46 to 5.16)	0.49		
Left side	0.73 (0.27 to 2.02)	0.55		
BMI	1.18 (1.05 to 1.31)	0.004*	1.19 (1.06 to 1.34)	0.004*
ASA grade 4	4.98 (1.72 to 14.43)	0.003*	5.67 (1.68 to 19.07)	0.005*
Diabetes	2.58 (0.92 to 7.23)	0.07		
Stroke	0.31 (0.04 to 2.48)	0.27		
Cardiac disease	0.82 (0.25 to 2.68)	0.74		
Renal failure	1.95 (0.57 to 6.64)	0.29		
TAD	1.07 (0.99 to 1.14)	0.08		
Calcar TAD	1.07 (1.00 to 1.15)	0.06		
CTI	0.12 (0.00 to 27.16)	0.44		
Canal diameter	1.07 (0.92 to 1.25)	0.40		
CFR-AP	0.16 (0.00 to 14.29)	0.43		
CFR-Lat	0.06 (0.00 to 11.51)	0.29		
NSA	0.93 (0.86 to 1.00)	0.06		
Lateral angulation	1.08 (0.99 to 1.17)	0.10	1.13 (1.02 to 1.25)	0.02*
Positive AMCS	0.31 (0.11 to 0.87)	0.03*	0.27 (0.08 to 0.88)	0.03*
Excellent CRQC	0.39 (0.14 to 1.10)	0.07		
Cement augmentation	0.99 (0.33 to 3.01)	0.99		

BMI, body mass index; ASA, American Society of Anesthesiologists; DM, diabetes mellitus; TAD, tip apex distance; Cal-TAD, calcar referenced tip apex distance; CTI, cortical thickness index; CFR-AP, canal fit ration in AP view; CFR-lat, canal fit ratio in lateral view; NSA, neck shaft angle; AMCS, anteromedial cortical support; CRQC, Chang reduction quality criteria

*Significant diffence with *p* < 0.05

## Discussion

The treatment of basicervical femoral neck fractures (BFNF) in the elderly is still a challenging issue regarding to the diagnosis and surgical options. The present study aimed to identify the risk factors for implant-related complication in the geriatric patients sustained BFNF and treated with PFNA. Our findings showed that the reoperation rate was 7.4%, comparable to the previous systematic review by Dekhne et al. as a prevalence of 8% revision rate after cephallomedullay nail [28]. Moreover, The results of this study also showed that the reoperation rate in Mechanical complication group was significantly higher than those in No implant failure group (47 vs. 2%, *p* < 0.001), and therefore, highlighted the influence on the postoperative morbidity influenced by the failure of PFNA fixation after BFNF.

Regarding to the predicting factors for implant-related complication after PFNA in BFNF, our study revealed that higher BMI, ASA grade 4, higher lateral angulation, and negative AMCS were all significantly associated with implant failure (Table 3). The effect from higher BMI on the PFNA failure could be explained by the increase of the overall loading on the implant in the reduced bone strength as in the geriatric patients with osteoporosis [29]. Also, the elderly patients with multiple comorbidities, as ASA grade 4, are commonly associated with severe osteoporosis due to the overall poor health condition and directly correlated with the poor surgical outcomes in many previous studies on PFNA fixation [30, 31]. In terms of predicting factors as lateral angulation and negative AMCS, the results of the present study were comparable to those in previous studies [32, 33]. These findings underscore the importance of meticulous surgical technique in achieving optimal outcomes in the geriatric patients with BFNF and treated with PFNA.

This study also has some limitations. Firstly, the retrospective design led to some incomplete medical records resulting in information bias. Secondly, the sample size in this study was relatively small, and therefore limiting the power to find the other significant independent risk factors, such as, diabetes, TAD, Cal-TAD, blade position, NSA, or CRQC. Recently, Yang et al. [34] published a finite element analyis study of the implant selection and screw positioning in BFNFs, demonstrating that the inferior position of hip screw or blade provides better biomechanical advantages the central position, by lowering femoral head displacement and maximum implant stress [34]. These findings suggest that there might be other significant risk factors for PFNA-related complications in the treatment of BFNFs. Therefore, larger study utilizing multicenter data are needed to identify these potential risk factors. Lastly, defining excessive sliding as more than 10 mm may have influenced the results; future studies might consider using a threshold of 15 mm to better evaluate fixation failure.

## Conclusion

The operative treatment in the elderly patients sustained BFNF with PFNA is generally safe with a low reoperative rate as 7.4%. However, the patients’ characteristics and some perioperative surgical factors—as high BMI, ASA grade 4, and poor reduction quality with increased lateral angulation and negative anteriomedial cortical support—could affect the surgical outcome and resulting in the implant-related complication. Therefore, comprehensive preoperative assessment and meticulous surgical technique with tailored postoperative care are needed to improve the outcomes for these patients. Further research is needed to validate these findings and explore the impact of targeted interventions on reducing fixation failure rates.

## Data Availability

The data that support the findings of this study are not openly available due to reasons of sensitivity and are available from the corresponding author upon reasonable request.

## References

[1] Chen CY, Chiu FY, Chen CM, Huang CK, Chen WM, Chen TH (2008) Surgical treatment of basicervical fractures of femur—a prospective evaluation of 269 patients. J Trauma 64(2):427–429 18301209 10.1097/01.ta.0000239255.47280.6f

[2] Yoo JI, Cha Y, Kwak J, Kim HY, Choy WS (2020) Review on basicervical femoral neck fracture: definition, treatments, and failures. Hip Pelvis 32(4):170–181 33335865 10.5371/hp.2020.32.4.170 PMC7724026

[3] Imren Y, Gurkan V, Bilsel K, Desteli EE, Tuna M, Gurcan C et al (2015) Biomechanical comparison of dynamic hip screw, proximal femoral nail, cannulated screw, and monoaxial external fixation in the treatment of basicervical femoral neck fractures. Acta Chir Orthop Traumatol Cech 82(2):140–144 26317185

[4] Sundkvist J, Hulenvik P, Schmidt V, Jolback P, Sundfeldt M, Fischer P et al (2024) Basicervical femoral neck fractures: an observational study derived from the Swedish Fracture Register. Acta Orthop 95:250–255 38775110 10.2340/17453674.2024.40503 PMC11109924

[5] Lee YK, Yoon BH, Hwang JS, Cha YH, Kim KC, Koo KH (2018) Risk factors of fixation failure in basicervical femoral neck fracture: which device is optimal for fixation? Injury 49(3):691–696 29433801 10.1016/j.injury.2018.02.009

[6] Yoo J, Kim S, Choi J, Hwang J (2019) Gamma 3 U-Blade lag screws in patients with trochanteric femur fractures: are rotation control lag screws better than others? J Orthop Surg Res 14(1):440 31842911 10.1186/s13018-019-1427-z PMC6916220

[7] Davis RA, Henningsen JD, Huff S, Schneider AD, Hijji FY, Froehle A et al (2022) Primary hemiarthroplasty for the treatment of basicervical femoral neck fractures. Cureus 14(5):e25210 35746995 10.7759/cureus.25210 PMC9211754

[8] Guo J, Dong W, Jin L, Yin Y, Zhang R, Hou Z et al (2019) Treatment of basicervical femoral neck fractures with proximal femoral nail antirotation. J Int Med Res 47(9):4333–4343 31327294 10.1177/0300060519862957 PMC6753548

[9] Watson ST, Schaller TM, Tanner SL, Adams JD, Jeray KJ (2016) Outcomes of low-energy basicervical proximal femoral fractures treated with cephalomedullary fixation. J Bone Joint Surg Am 98(13):1097–1102 27385683 10.2106/JBJS.15.01093

[10] Jamal Sepah Y, Umer M, Khan A, Ullah Khan Niazi A (2010) Functional outcome, mortality and in-hospital complications of operative treatment in elderly patients with hip fractures in the developing world. Int Orthop 34(3):431–435 19471932 10.1007/s00264-009-0803-4 PMC2899302

[11] Mereddy P, Kamath S, Ramakrishnan M, Malik H, Donnachie N (2009) The AO/ASIF proximal femoral nail antirotation (PFNA): a new design for the treatment of unstable proximal femoral fractures. Injury 40(4):428–432 19230885 10.1016/j.injury.2008.10.014

[12] Liu Y, Tao R, Liu F, Wang Y, Zhou Z, Cao Y et al (2010) Mid-term outcomes after intramedullary fixation of peritrochanteric femoral fractures using the new proximal femoral nail antirotation (PFNA). Injury 41(8):810–817 20472234 10.1016/j.injury.2010.03.020

[13] Kammerlander C, Gebhard F, Meier C, Lenich A, Linhart W, Clasbrummel B et al (2011) Standardised cement augmentation of the PFNA using a perforated blade: A new technique and preliminary clinical results. A Prospect Multicentre Trial Injury 42(12):1484–1490 10.1016/j.injury.2011.07.010 21855063

[14] Goodnough LH, Wadhwa H, Tigchelaar SS, DeBaun MR, Chen MJ, Graves ML et al (2022) Indications for cement augmentation in fixation of geriatric intertrochanteric femur fractures: a systematic review of evidence. Arch Orthop Trauma Surg 142(10):2533–2544 33829301 10.1007/s00402-021-03872-6

[15] Yeganeh A, Abbasi M, Gorgani H-o-l, Moghtadaei M (2019) Plate augmentation for nonunion of femoral shaft fractures. Shafa Orthopedic Journal. In Press.

[16] Baumgaertner MR, Curtin SL, Lindskog DM, Keggi JM (1995) The value of the tip-apex distance in predicting failure of fixation of peritrochanteric fractures of the hip. J Bone Joint Surg Am 77(7):1058–1064 7608228 10.2106/00004623-199507000-00012

[17] Kashigar A, Vincent A, Gunton MJ, Backstein D, Safir O, Kuzyk PR (2014) Predictors of failure for cephalomedullary nailing of proximal femoral fractures. Bone Joint J 96(8):1029–1034 25086117 10.1302/0301-620X.96B8.33644

[18] Cleveland M, Bosworth DM, Thompson FR, Wilson HJ Jr, Ishizuka T (1959) A ten-year analysis of intertrochanteric fractures of the femur. J Bone Joint Surg Am 41:1399–1408 13849408

[19] Baumgärtner R, Heeren N, Quast D, Babst R, Brunner A (2015) Is the cortical thickness index a valid parameter to assess bone mineral density in geriatric patients with hip fractures? Arch Orthop Trauma Surg 135(6):805–810 25801811 10.1007/s00402-015-2202-1

[20] Nguyen BN, Hoshino H, Togawa D, Matsuyama Y (2018) Cortical thickness index of the proximal femur: a radiographic parameter for preliminary assessment of bone mineral density and osteoporosis status in the age 50 years and over population. Clin Orthop Surg 10(2):149–156 29854337 10.4055/cios.2018.10.2.149 PMC5964262

[21] Ergisi Y, Ozdemir E, Korkmazer S, Kekec H, Altun O, Yalcin N (2022) What is the importance of distal nail diameter in the treatment of intertrochanteric femur fractures? Jt Dis Relat Surg 33(3):639–644 36345193 10.52312/jdrs.2022.850 PMC9647669

[22] Boese CK, Dargel J, Oppermann J, Eysel P, Scheyerer MJ, Bredow J et al (2016) The femoral neck-shaft angle on plain radiographs: a systematic review. Skeletal Radiol 45(1):19–28 26305058 10.1007/s00256-015-2236-z

[23] Tufescu T, Sharkey B (2013) The lateral radiograph is useful in predicting shortening in 31A2 pertrochanteric hip fractures. Can J Surg 56(4):270–274 23883498 10.1503/cjs.007412 PMC3728247

[24] Mao W, Liu CD, Chang SM, Yang AL, Hong CC (2024) Anteromedial cortical support in reduction of trochanteric hip fractures: from definition to application. J Bone Joint Surg Am 106(11):1008–1018 38683886 10.2106/JBJS.23.01023

[25] Mao W, Ni H, Li L, He Y, Chen X, Tang H et al (2019) Comparison of Baumgaertner and Chang reduction quality criteria for the assessment of trochanteric fractures. Bone Joint Res 8(10):502–508 31728190 10.1302/2046-3758.810.BJR-2019-0032.R1 PMC6825041

[26] Wild M, Jungbluth P, Thelen S, Laffree Q, Gehrmann S, Betsch M et al (2010) The dynamics of proximal femoral nails: a clinical comparison between PFNA and Targon PF. Orthopedics. 10.3928/01477447-20100625-04 20704115 10.3928/01477447-20100625-04

[27] Zhang S, Wang R, Li J, Li C, Wang T, Yang Y et al (2025) Risk factors of excessive sliding in elderly patients with intertrochanteric fractures treated with PFNA-II: A retrospective observational study. BMC Musculoskelet Disord 26(1):255 40087659 10.1186/s12891-025-08479-1 PMC11907781

[28] Dekhne MS, Thomas HM, Haider T, Mortensen S, Rodriguez EK, Weaver MJ et al (2021) Treatment and outcomes of basicervical femoral neck fractures: a systematic review. J Orthop Surg (Hong Kong) 29(1):23094990211003344 33779387 10.1177/23094990211003344

[29] Konstantinidis L, Papaioannou C, Blanke P, Hirschmuller A, Sudkamp NP, Helwig P (2013) Failure after osteosynthesis of trochanteric fractures. Where is the limit of osteoporosis? Osteoporos Int 24(10):2701–2706 23702701 10.1007/s00198-013-2392-8

[30] Kim JL, Jung JS, Kim SJ (2016) Prediction of ambulatory status after hip fracture surgery in patients over 60 years old. Ann Rehabil Med 40(4):666–674 27606273 10.5535/arm.2016.40.4.666 PMC5012978

[31] Yeoh CJ, Fazal MA (2014) ASA grade and elderly patients with femoral neck fracture. Geriatr Orthop Surg Rehabil 5(4):195–199 26246942 10.1177/2151458514560471 PMC4252162

[32] Hong SH, Yu KH, Han SB (2024) Intramedullary impaction of the basicervical component is determinant of fixation failure in a simple two-part pertrochanteric fracture. J Orthop Trauma 38(4):220–226 38241062 10.1097/BOT.0000000000002770

[33] Li SJ, Chen SY, Chang SM, Du SC, Hu SJ (2023) Insufficient proximal medullary filling of cephalomedullary nails in intertrochanteric femur fractures predicts excessive postoperative sliding: a case-control study. BMC Musculoskelet Disord 24(1):156 36855090 10.1186/s12891-023-06213-3 PMC9972673

[34] Yang T, Noraddin F, Liu B, Zhang Z, Gu HL (2025) Finite element analysis of implant selection and screw positioning in proximal femoral basicervical fractures. Sci Rep 15(1):35360 41068282 10.1038/s41598-025-19260-8 PMC12511412

